# New products

**DOI:** 10.1155/S1463924697000229

**Published:** 1997


Journal of Automatic Chemistry, Vol. 19, No. 5 (September–October 1997) pp. 187–191

### New products

### Automated synthesis

Labcaire has been selected by the Technology Partnership (TTP), to integrate a modified FE4850 with their Myriad synthesizer to produce a self-contained synthesis workstation. Myriad will provide research scientists with a powerful, flexible tool for drug development and discovery. TTP will use Labcaire's FE4850 filtration fume cabinet for the efficient and safe containment of hazardous chemical fumes. The custom-built cabinet is to be combined with TTP's automated chemistry synthesis system, which the company will market to the pharmaceutical industry worldwide.

*More information from Labcaire Systems Ltd, 15 Hither Green, Clevedon, Somerset BS21 6XU, UK. Tel.: 01275 340033; fax: 01275 341313.*

### Colour QC

An easy-to-use quality control software has been launched by Macbeth. Called Optiview Lite, it is a good entry-level option for new users or companies who require only basic but highly accurate colour control. Optiview Lite can be efficiently used with minimal training or knowledge of colour science. The operator measures or recalls a standard or trial; views the results graphically or numerically; and then saves or prints the result. The system can be used with either a keyboard or a mouse.

Optiview Lite can be used with any of Macbeth's high precision Color-Eye range of benchtop and portable spectrophotometers.

*Details from Macbeth, Macbeth House, Pacific Road, Altrincham, Cheshire WA14 5BJ, UK. Tel.: 0161 926 9822; fax: 0161 926 9835.*

### 1996 Metrology for World Class Manufacturing Awards

P S Analytical has won the Category 3 (one of four groups) awards for Measurement for Manufacturing Excellence for its submission on 'Improvements relating to the analysis of low levels of mercury in the environmental and petrochemical market areas'. P S Analytical Ltd specializes in the ultra low level detection of mercury, arsenic, selenium, antimony, tellurium and bismuth. Founded by Professor Peter B. Stockwell in 1983, PSA has gained international recognition leading the field of detection of environmentally important elements in water, soils, sludges, effluents and gases and has customer installations worldwide.

Since 1988, PSA has established a global reputation as the experts in mercury analysis, through linking expertise in sample preparation using vapour generation techniques to the design of a specific sensitive detector for mercury measurements based on atomic fluorescence. The PSA Merlin Atomic Fluorescence Detector was originally developed for the UK water industry to meet EC legislative requirements in 1988. By offering quantitation, accurate over six orders of magnitude at parts per billion (ppb) levels, this detector has found an extensive range of applications.

P S Analytical finds unequivocable analytical solutions for industry by researching and developing new applications then manufacturing and distributing the products. Four of P S Analytical's staff are currently involved in research programmes leading to PhDs in association with Professor Les Ebdon and the Department of Environmental Sciences at the University of Plymouth.

*For more information contact Professor Stockwell at P S Analytical Ltd, Arthur House, Unit 3, Crayfields Industrial Estate, Main Road, St Pauls Cray, Orpington, Kent BR5 3HP, UK. Tel.: 01689 891211; fax: 01689 896009.*

### Microwave digestion system

The Multiwave is a versatile and powerful microwave sample digestion system for high pressure acid digestion of all types of organic and inorganic samples. It is a closed system, with six-vessel capacity, unique pressure and temperature monitoring system and multi-level safety system to ensure minimum digestion times and maximum digestion efficiency. Of particular benefit is the ability to digest different sample types simultaneously. Each vessel can be separately temperature controlled to ensure optimum digestion conditions.

Major applications of the Multiwave include wet chemical pressurized digestions of numerous sample types for elemental trace analysis using AAS, ICP-OES, ICP-MS and electrochemical techniques. Used for environmental samples, liquid samples with a high organic content, food, chemicals, pharmaceuticals, petrochemical and geological materials, the Multiwave can significantly reduce sample preparation time.

*For further information, contact Perkin-Elmer Ltd, Post Office Lane, Beaconsfield, Buckinghamshire HP9 1 QA, UK. Tel.: 01419 676161; fax: 01494 679331/3; internet: http://www.perkin-elmer.com.*

### Electrochemistry

Solartron has redefined the price/performance of potentiostats with the introduction of the 1285. Designed to fill the gap between ultra-high accuracy instruments and simple bench-top units, the new interface offers top-end laboratory performance at a highly aggressive price; the interface is supplied with CorrWare software.

DC analysis is facilitated by the 1285's measurement precision, stability and 1 microvolt/1 picoamp resolution, enabling studies into corrosion coating and inhibitor performance, where resistance can exceed 1 Gohm.

0142-0453/97 $12·00 © 1997 Taylor & Francis Ltd

Equally, the potentiostat's ±14·5 V range simplifies examination of complete accumulators, including the behaviour of multi-cell structures. The 1285 provides three separate ohmic-drop compensation techniques for investigations using high impedance electrolyte solutions.

The 1285 can run stepped or smooth polarization sweeps—a requirement of specifications such as ASTM G5—without the need for additional equipment. A built-in sweep freeze capability eliminates the effects of cell disturbance between tests. The 1285 offers a true analogue sweep, not a digital approximation. Sweeps can be set up in both potentiostat and galvanostat mode.

Electrochemical noise, being entirely passive, is one of the most realistic corrosion investigation techniques available today. One of its primary applications is to detect the onset and advancement of pitting corrosion. Fundamental to this technique is the requirement to monitor extremely small voltage and current fluctuations simultaneously over a period of time without applying any external stimulus. Dual, high accuracy, five-digit voltmeters provide the 1285's voltage/current measurement with μV/pA- resolution, offering unrivalled electrochemical noise measurement.

Complete flexibility of interconnection is provided by the 1285: high-accuracy four-terminal 'driven-shield' connection for biological/biomedical studies of ion transport across a membrane to assess drug osmosis rates, membrane pore size or tissue healing rates following drug introduction; three-terminal for linear polarization resistance measurements of oil pipelines and general corrosion experiments; and two-terminal interfacing for general electrochemistry work. All connections are fully floating, enabling the instrument to provide high resolution measurements both in the field, on grounded structures such as storage tanks and pipelines, and in the laboratory, when using such equipment as autoclaves which are grounded for safety reasons.

*For further information contact Pauline Taylor, Solartron Ltd, Victoria Road, Farnborough GU14 7PW, UK. Tel.: 01252 376666; fax: 01252 543854; email: taylor@solartron.com; web site: www.solartron.com.*

### PS Analytical staff receive awards

At the recent Biennial National Atomic Spectroscopy Symposium Conference at the University of East Anglia, P S Analytical staff were honoured with two awards. Dr Warren Corns won the Hilger Spectroscopy Prize to mark his contribution to spectroscopic research. Dr Corns presented a poster display which illustrated the publications for which he has been primarily responsible. The foundation for this programme was laid down during a PhD programme at Plymouth University with Professor Les Ebdon and Steve Hill, in association with Professor Peter Stockwell from PSA. Dr Corns' current research involves specialist applications in the natural gas and stack monitoring fields for mercury analysis and a programme of research relating to speciation studies of mercury, arsenic and selenium.

The second award, to Jameel Mohammed, a PSA/Plymouth PhD student, was for the best student poster display. The work was on the analysis of sodium bisulphate using a vapour generation approach; this analyte is important in the caustic soda industries.

### Combinatorial chemistry

The HP1100 combinatorial chemistry system combines the HP 1100 Series liquid chromatography (LC) modules with a Gilson 233 XL sampling injector, HP ChemStation for LC systems and dedicated software to provide a single point for sample analysis, fractionation and data management. The user has a choice of highly reliable modules from the HP 1100 Series LC, as well as a range of versatile detectors, including a variable wavelength detector, diode array detector and a recently introduced mass-selective detector (HP 1100 Series LC/MSD).

The standard HP ChemStation has been extended with dedicated software that runs within the Microsoft Windows 95 and NT environments. This software fulfils the special requirements of combinatorial-chemistry analysis by providing control of up to six 96-well plates for sampling and fraction-collection, along with colour-coded, graphic sample tracking and data evaluation in a single screen. Each peak of a sample is labelled, showing a correlation between the fraction position and the original sample. In addition, sample selection is easier because the user has the choice of random sampling and/or sampling by row and column.

The software has two modes. In the sampler mode, samples can be injected from each of the 96-well plates and analysed. In the sampler/fraction collector mode, the sampler can take samples from each of the 96-well plates and collect fractions into other 96-well plates. The sampler mode is specifically designed for high-throughput analysis.

*Details from Hewlett-Packard Europe, Chemical Analysis Group, 150 route du Nant-d'Avril, CH 1217 Meyrin 2, Switzerland. Tel.: 4122 780 82 27; fax: 4122 780 83 21.*

### Server

Hewlett-Packard's ChemAccess client/server software offers a single approach to instrument control, data evaluation, reporting and automation. The server can be combined with the HP ChemStations for gas chromatography, liquid chromatography and capillary electrophoresis to offer centralized instrument control, allowing laboratory staff to control and monitor the status of instruments from a desktop PC. It offers the following additional benefits over standard server software: the overall status of all laboratory instruments can be accessed directly from any remote PC linked to the server; the status of individual laboratory instruments can be monitored; real time plots can be displayed; sequence and method control; remote macro execution, and data organisation tools. These features eliminate the need for staff to check each instrument manually. Since remote run control and status information can be undertaken, such as stopping a sample run, if necessary, staff do not waste time or samples unnecessarily.

By allowing users to print data from other PCs linked to the server, the HP ChemAccess Client/Server also frees

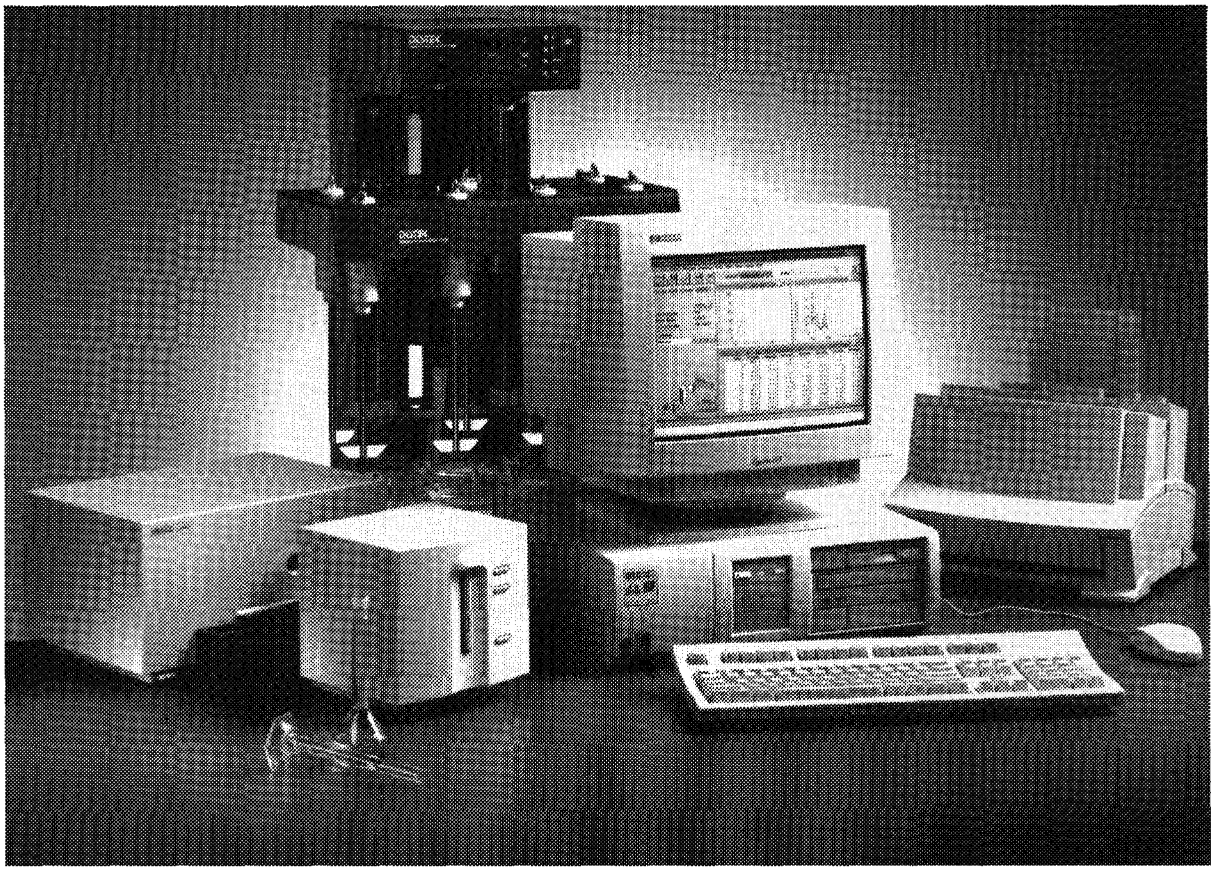

*The HP 8453 which is one of Hewlett-Packard Europe's dissolution testing systems based on the HP 8453 UV-VIS spectrophotometer and HP ChemStation for UV-VIS spectroscopy. These new systems fully integrate third-party equipment by providing, for example, full control through the HP ChemStation of dissolution baths from all major manufacturers.*

*Details from Hewlett-Packard Europe, Chemical Analysis Group, 150, route du Nant-d'Avril, CH-1217 Meyrin 2, Switzerland.*

laboratory instruments from routine printing. This ensures that they can be dedicated to sample processing, increasing throughput. Printers can be located anywhere on the network, which saves on bench space.

In addition, the HP ChemAccess Client/Server provides added security: logon and passwords provide secure access to the network and server resources. Predefined 'permissions' determine user access and ability to modify files, ensuring that precious programs and date files are protected from accidentally being overwritten.

The HP ChemAccess Client/Server makes file back-up easy: one person can easily back-up all HP ChemStation files using the server itself.

*Details from Hewlett-Packard (as above).*

### Thermostatted autosampler

The thermostatted autosampler is an addition to the HP 1100 Series of liquid chromatography (LC) modules and can be controlled by an HP ChemStation for LC or by the handheld HP 1100 Series control module. It uses Peltier elements for efficient air cooling, and the cooled air is kept dry, eliminating condensation. Specially designed sample trays ensure effective temperature control, regardless of how many vials are in the tray. The sample trays are removable, providing for easy handling of large numbers of sample—for example, transport from refrigerator. Clock-time programming enables the temperature control to be turned on or off for optimized energy consumption. The temperature control can be turned off sequence of analyses.

*Details from Hewlett-Packard (as above).*

### Rhône-Poulenc Rorer announces opening of new pilot plant in Dagenham, UK

The pharmaceutical company, Rhône-Poulenc Rorer (RPR)'s new £17m pilot plant in Dagenham, UK was opened at the end of April 1997. This new facility has been built for the preparation of innovative new compounds undergoing pre-clinical and clinical evaluation. The plant was designed to be fully compliant with current Good Manufacturing Practice (CGMP) standards. RPR has made every effort to ensure the plant was designed in such a way that there will be no adverse impact on the environment.

Also at the end of April 1997, RPR announced the creation of a combined venture with Cambridge University and Imperial College, London to use advanced computing and combinatorial chemistry to discover innovative drugs capable of treating previously incurable conditions. RPR will invest £4m over three years in the venture, which is named TeknoMed.

RPR launched two new drugs in the UK during 1996: Rilutek® (riluzole), the first treatment for motor neurone disease and Taxotere® (docetaxel), a treatment for advanced breast cancer.

*For further details contact Rhône-Poulenc Rorer, 50 Kings Hill Avenue, Kings Hill, West Malling, Kent ME19 4AH, UK. Tel.: 01732 584000; fax: 01732 584080.*

### EDXRF spectrometer

Oxford Instrument's Industrial Analysis Group has been awarded US patent #5528647 for the technology of the Lab-X3000, its benchtop EDXRF (Energy Dispersive X-ray Fluorescence) spectrometer. This analyser has a separate autosampler and is the latest in Oxford's Lab-X series. It uses Oxford's *Focus-5* technology to increase sensitivity and can be used in many different industries. It is capable of detecting elements from aluminium to uranium, from ppm to high %. However, it does not use radio-isotopes, hazardous chemicals, nor complex dilutions.

Several dedicated applications packages are also available which cover common uses such as the elemental analysis of cement, the determination of sulphur and other elements in petroleum products, iron in silica sand, salt in food products, antimony/bromine in polymers, or silicone coating on paper and films. Samples are analysed 'as is', with little or no preparation, in the form of solids, liquids, powders, papers, films, or pastes. Testing is usually complete in 60 seconds.

The autosampler allows for unattended operation for both routine analysis of samples and restandardization of a calibration line. Solid, liquid and powder analysis can be undertaken using Oxford sample holders combined with the corresponding sample tray. The 12-position, removable sample tray, robotic arm for transfer of samples and associated electronics control module can easily be operated by both lab and non-lab staff in quality control or research and development. The unit is situated on top of the Lab-X3000 and does not require additional benchspace.

*For further information contact Oxford Instruments' Industrial Analysis Group at: 130A Baker Avenue Ext., Concord, MA 01742, USA tel. 800 447 4717 or 508 371 9009 or 19/20 Nuffield Way, Abingdon, OXON OX14 1TX, UK. Tel.: 01235 532 123; fax 01235 535 416.*

### Quantity and quality with the new Quattro LC

Micromass is the Quattro LC compact triple stage quadrupole system that has been specifically optimized for API LC-MS-MS applications in the pharmaceutical industry, bioanalytical contract laboratories and clinical screening programmes. Loss of performance can occur due to a build-up of matrix material in the LC-MS interface, but this can be alleviated with Crossflow. Additionally, by enabling analyses with involatile mobile phase modifiers such as ion pairing reagents and phosphate buffers, Crossflow can save on method redevelopment time. Its use of a novel counter electrode in the API interface, designed to trap involatile material, allows the ion source to be used with dirty samples. An aerosol spray is formed, during operation, between the coaxial nebulizing probe and the rear face of the electrode. Involatile material becomes trapped in a deep well within the electrode and the periphery of the spray cone is sampled through a channel in the side of the well. Sampled material is subsequently blown out of the channel, forming a spray across the sampling cone. This then provides a division between the atmospheric pressure region of the source and the rough vacuum stage, with neutral material from the spray crossing harmlessly above the skimmer, and ions being deflected into the skimmer aperture by a voltage applied to the counter electrode and acting as a repeller.

With Quattro LC, there is the option to fully combine Waters Alliance or HP 1100 solvent delivery systems, Gilson autosamplers for 96-well microplates, Gilson solid phase extraction systems and a selection of UV-VIS detection options such as a diode array. For maximum throughput, a second HPLC may be optionally incorporated.

*A brochure is available from Micromass Limited, Floats Road, Wythenshawe, Manchester, M23 9LZ, UK. Tel: 161 945 4170; fax: 161 998 8915.*

### Platform LC

A new brochure from Micromass describes Platform LC, a simpler, more refined version of the Platform system. Designed for the liquid chromatographer, method development chemist and QA/QC analyst, this new system features proven Atmospheric Pressure Ionization (API) and is fully compatible with LC flow rates up to 2 ml/min and Micromass' proprietary CrossFlow™ technology. As a result, LC-MS with complex biological/environmental matrices and involatile mobile phase modifiers is possible. Even phosphate buffers and ion paring reagents can be transiently accommodated.

A unique mass spectral 'fingerprint' is revealed for each eluting compound when Electrospray and APcl LC-MS techniques are used with the Platform LC. The resulting 'orthogonal' MS information is then handled similarly to data obtained from an LC separation with diode-array UV-VIS detection. Spectra can be combined and background subtracted to determine the molecular weight of each analyte. Furthermore, detailed structural information may be inferred from the fragmentation pattern optionally available within a spectrum, and spectra may be library searched against user-defined libraries.

Platform LC has the capacity to be both a highly selective detector and a near universal detector, depending on which is most appropriate for the user's requirements. 'Mass chromatograms' can be plotted in real time, or post-acquisition, for a single mass, or a range of masses summed together. The new system also provides access to all the qualitive and quantitive information required in the laboratory through a single chromatogram.

*Copies of the brochure from Dr Mark McDowall, Micromass UK Ltd, Tudor Road, Altrincham, Cheshire, WA14 5RZ, UK. Tel.: 161 282 9666; fax: 161 282 4400; E-mail: mark.mcdowall@micromass.co.uk.*

### CONFERENCES

### 20th International Symposium on Capillary Chromatography, May 25-29, 1998. Palazzo dei Congressi, Riva del Garda, Italy

The 20th International Symposium on Capillary Chromatography will take place in Riva del Garda, Italy from 25 to 29 May 1998.

*First curriculars are now available from Professor Dr P. Sandra, Symposium Chairman, 10PMS, Kennedy Park 20, B 8500 Kortrijk, Belgium. Tel.: 32 56 204 960.*

### Winter Plasma Conference 1998

*For further details please contact Dr Ramon Barnes at the Department of Chemistry, Lederle GRC Towers, University of Massachussetts, Box 34510, Amherst, MA 01003-4510 USA.*

